# Severity and prognostic factors of SARS-CoV-2-induced pneumonia: The value of clinical and laboratory biomarkers and the A-DROP score

**DOI:** 10.3389/fmed.2022.920016

**Published:** 2022-07-22

**Authors:** Miklós Szabó, Zsófia Kardos, Csaba Oláh, Péter Tamáska, Katalin Hodosi, Eszter Csánky, Zoltán Szekanecz

**Affiliations:** ^1^Department of Pulmonology, Borsod Academic County Hospital, Miskolc, Hungary; ^2^Department of Rheumatology, Borsod Academic County Hospital, Miskolc, Hungary; ^3^Faculty of Health Sciences, University of Miskolc, Miskolc, Hungary; ^4^Department of Neurosurgery, Borsod Academic County Hospital, Miskolc, Hungary; ^5^Department of Radiology, Borsod Academic County Hospital, Miskolc, Hungary; ^6^Department of Rheumatology, Faculty of Medicine, University of Debrecen, Debrecen, Hungary

**Keywords:** COVID-19, tocilizumab (IL-6 inhibitor), prognosis, pneumonia, outcome, A-DROP score

## Abstract

**Introduction:**

Numerous clinical and laboratory scores that include C-reactive protein (CRP), D-dimer, ferritin, lactate dehydrogenase (LDH), interleukin 6 (IL-6), procalcitonin (PCT), blood urea nitrogen (BUN), creatinine levels and oxygenation (PaO_2_ and SaO_2_) have been used for the prognosis of COVID-19. In addition, composite scores have been developed for the assessment of general state and risk in community-acquired pneumonia (CAP) that may be applied for COVID-19 as well. In this study, we assessed severity and potential prognostic risk factors for unfavorable outcome among hospitalized COVID-19 patients. We also applied the A-DROP general scoring system used in CAP to COVID-19.

**Patients and methods:**

Altogether 233 patients admitted to our center with COVID-19 were included in the study. Clinical status, several laboratory biomarkers described above, indicators of oxygenation were determined at hospital admission. We also applied the A-DROP composite scoring system that includes Age (≥ 70 years in males and ≥ 75 years in females), Dehydration (BUN ≥ 7.5 mmol/l), Respiratory failure (SaO_2_ ≤ 90% or PaO_2_ ≤ 60 mmHg), Orientation disturbance (confusion) and low blood Pressure (systolic BP ≤ 90 mmHg) to COVID-19.

**Results:**

At the time of admission, most patients had elevated CRP, LDH, ferritin, D-dimer, and IL-6 levels indicating multisystemic inflammatory syndrome (MIS). Altogether 49 patients (21.2%) required admission to ICU, 46 (19.7%) needed ventilation and 40 patients (17.2%) died. In the binary analysis, admission to ICU, the need for ventilation and death were all significantly associated with the duration of hospitalization, history of hypertension or obesity, confusion/dizziness, as well as higher absolute leukocyte and neutrophil and lower lymphocyte counts, elevated CRP, PCT, LDH, ferritin, IL-6, BUN, and creatinine levels, low PaO_2_ and SaO_2_ and higher A-DROP score at the time of admission (*p* < 0.05).

**Conclusion:**

Numerous laboratory biomarkers in addition to obesity, dizziness at the time of admission and the history of hypertension may predict the need for ICU admission and ventilation, as well as mortality in COVID-19. Moreover, A-DROP may be a suitable scoring system for the assessment of general health and disease outcome in COVID-19.

## Introduction

In late 2019, a new strain of β coronavirus called severe acute respiratory syndrome coronavirus 2 (SARS-CoV-2) was identified in Wuhan, China, which caused a worldwide epidemic due to its rapid spread (1, 2). The COVID-19 pandemic puts an extreme load on healthcare systems including intensive care units (ICU) all over the world (1, 3–5). In the first waves of the epidemic, the hospitalization rate was 5–10 percent, while global mortality was 2–3 percent. In the majority of patients, the disease is asymptomatic or mild, but in some patient groups it may be severe with potentially fatal outcome (6). SARS-CoV-2 virus-induced pneumonia is a part of multisystemic inflammatory syndrome (MIS) associated with the advanced stages of COVID-19. MIS often leads to the damage of multiple organs and death (3, 4, 7).

The initial assessment of the severity of community-acquired pneumonia (CAP) is important for patient management (8). In addition the number of patients diagnosed with COVID-19 pneumonia in this epidemic is high, while health resources are finite. Identification of high risk patients are of paramount importance for the optimal use of hospital capacity and patient safety. There have been attempts to identify prognostic factors that might predict the outcome of early SARS-CoV-2 infection and COVID-19-associated pneumonia (4, 9–11). Comorbidities, such as hypertension, hyperlipidemia, ischemic heart disease, congestive heart failure, chronic pulmonary disease, diabetes mellitus, cerebrovascular disease, dementia, liver disease, chronic kidney disease, malignancies, sickle cell disease, organ transplantation, and other immunocompromising conditions have been associated with a higher risk of severe disease and death (12–15). Symptoms including dyspnea, coughs, expectoration, hemoptysis, abdominal pain, anorexia, diarrhea, fatigue, myalgia, arthralgia, and fever have been reported more common in severe than in mild COVID-19 patients (16). Physical examination provides valuable information about a patient’s severity and prognosis. Tachypnea, tachycardia, hypotension, hypoxemia, confusion observed on physical examination are poor prognostic signs in COVID-19 patients (17, 18).

Laboratory tests are essential to determine hospitalization and therapy in patients with symptoms of infection. Several laboratory parameters monitoring hematological status or biochemical, inflammatory, immunological, and coagulation processes have been identified as prognostic factors for COVID-19 disease. Severe and fatal cases tended to show higher white blood cell, lower lymphocyte and platelet count, lower percentages of monocytes, eosinophils, and basophils, higher leukocyte and neutrophil-counts and a higher neutrophil lymphocyte ratio compared to mild cases (19, 20).

Some laboratory biomarkers including C-reactive protein (CRP), interleukin 6 (IL-6), ferritin, D-dimer, lactate dehydrogenase (LDH), leukopenia and cardiac troponin (cTn), in addition to clinical symptoms, such as fever have been identified as markers of MIS and cytokine storm associated with COVID-19 (21–23). For example, both CRP and D-dimer levels were elevated in patients in need for transfer to ICU compared to non-ICU patients (22). D-dimer > 3,500 ng/ml was associated with poor survival (24). Procalcitonin is a reliable indicator of bacterial co- or superinfection, the latter being a characteristic factor in the mortality of respirated patients (15).

Several composite scores have been developed for the assessment of general state and risk in CAP that may be applied for COVID-19 as well (25). These include APACHE, qSOFA, PSI, CURB65, and A-DROP (25). Among these scoring systems, CURB65 [confusion, blood urea nitrogen (BUN) > 7 mmol/l, respiratory rate ≥ 30/min, low blood pressure (BP; diastolic BP ≤ 60 mmHg or systolic BP < 90 mmHg) and age ≥ 65 years] has been introduced by the British Thoracic Society (8, 25, 26). More recently, A-DROP, a modified version of CURB65 has been validated by the Japanese Respiratory Society (8, 25). The A-DROP scoring system includes Age (≥70 years in males and ≥ 75 years in females), Dehydration (BUN ≥ 7.5 mmol/l), Respiratory failure (SaO_2_ ≤ 90% or PaO_2_ ≤ 60 mmHg), Orientation disturbance (confusion) and low blood Pressure (systolic BP ≤ 90 mmHg (8, 25). It has been confirmed that A-DROP and CURB65 are equivalent for predicting CAP severity (8, 25). The prognostic value of A-DROP has been studied in only very few cohorts (25, 27, 28).

In this study, we assessed severity and potential prognostic risk factors for unfavorable outcome among hospitalized COVID-19 patients admitted to our center. We also applied the A-DROP general scoring system used in CAP to COVID-19.

## Patients and methods

### Study design and patients

This single-center, retrospective cohort study was conducted at the dedicated COVID-19 department of the Borsod Academic County Hospital, Miskolc, Hungary. Data from patients hospitalized for COVID-19 pneumonia between October 1, 2020, and March 31, 2021 were retrospectively analyzed. Confirmation of SARS-CoV-2 infection was performed by RT-PCR method from throat-swab specimens. Pneumonia was confirmed by radiological imaging performing chest CT in 227 and plain X-ray in 6 cases. Most patients received favipiravir, corticosteroid (dexamethasone or methylprednisolone), enoxaparine treatment, as well as oxygen supplementation. In selected cases, remdesivir or tocilizumab was also introduced. The clinical criteria for hospital discharge included absence of fever for at least 3 days, cessation or significant improvement of respiratory symptoms, as well as clear improvement of the radiological picture.

The Ethics Committee of the Borsod Academic County Hospital approved this study (BORS 04/2021). We conducted this study according to the Declaration of Helsinki.

### Clinical, laboratory and imaging data collection

We reviewed all clinical electronic medical records and laboratory reports, as well as chest CT and X-ray images. We collected data on age, sex, as well as history of smoking, chronic comorbidities including hypertension, coronary arterial disease (CAD), chronic obstructive pulmonary disease (COPD) or bronchial asthma, previous stroke, diabetes mellitus, current malignancy, chronic kidney disease (CKD), obesity, as well as the use of systemic immunosuppressive therapy within 1 month prior to the analysis. We also recorded the duration and type of symptoms (fever: axillary temperature ≥ 38°C, cough, dyspnea, confusion), vital signs (blood pressure, oxygen saturation [SaO_2_] by pulse oximetry), laboratory values [white blood cell, absolute lymphocyte and platelet counts, serum CRP, ferritin, IL-6, LDH, D-dimer, procalcitonin (PCT), BUN, creatinine, alanine aminotransferase (ALT), aspartate aminotransferase (AST), D-dimer], partial arterial oxygen pressure (PaO_2_) as determined by blood gas analysis, as well as treatment (corticosteroids, antiviral, and antibacterial agents, targeted therapies) at hospital admission and during the time of hospitalization. We also recorded the occurrence of pulmonary embolism and Clostridium difficile infection during hospitalization. A-DROP scores were calculated from the data obtained at the time of hospital admission (8).

All data were evaluated by two physicians (MS, ZK) and a third researcher (ZS) adjudicated any difference in interpretation between the two primary reviewers.

### Outcome parameters

The primary outcome parameters were the need for intensive care, need for invasive (IV) vs. Non-invasive ventilation (NIV) and mortality. Mortality was calculated from mortality observed during hospitalization, and the disease-related mortality 30 days after discharge. The time of hospitalization was also recorded.

### Statistical analysis

Statistical analysis was performed using the SPSS software v.26.0 (IBM, Armonk, NY, United States). Data are expressed as mean ± *SD* for continuous and case number plus percentages (*n*, %) for categorical variables. The distribution of continuous variables was determined by Kolmogorov-Smirnov test. Continuous variables were assessed by Mann-Whitney *U*-tests. Nominal variables were compared by χ^2^ or Fisher’s exact test. Spearman’s analysis was used to test for correlations. Receiver Operating Characteristic (ROC) curves show the sensitivity and specificity for every possible cut-off for a test. Area under the ROC curve is measure of the usefulness of a characteristic, where a greater area means a more useful test. *P*-values < 0.05 were considered significant in all tests mentioned above.

## Results

### Characterization of patients

Altogether 233 patients were included in this study. Their main characteristics are included in Table 1. The patient cohort included 148 men and 85 women with a mean age of 56.8 ± 8.7 years (range: 40–76 years). Disease duration (time from the first symptom to hospital admission) was 8.5 ± 5.3 days (range: 1–35 days). Altogether 7.3% received immunosuppression, 19.1% were current smokers. The medical history of the patients included hypertension (65.1%), CAD (22%), stroke (6%), CKD (5.6%), diabetes mellitus (27.2%), obesity (30.6%), malignancies (4.3%), and COPD/asthma (22%). At the time of admission, about two-third of patients had fever, dyspnea and/or coughs, while 4.3% had confusion/dizziness (Table 1). According to the mean laboratory values, most of these patients had elevated CRP, LDH, ferritin, D-dimer and IL-6 levels indicating systemic inflammation (MIS) (Table 1). Out of the 233 hospitalized patients, 49 (21.2%) required admission to ICU. Altogether 46 patients (19.7%) needed ventilation, out of which 9 (3.9%) required NIV and 37 (15.9%) invasive ventilation (IV). Forty patients (17.2%) died. The mean (± *SD*) duration of hospitalization was 12.1 ± 6.8 days (range: 2–48 days) (Table 1).

**TABLE 1:** Patient characteristics.

Parameters at baseline	Total N	Mean ± *SD* or *N* (%)	Normal range
Age (years)	233	56.8 ± 8.7	–
Female: male ratio	233	85:148	–
Disease duration (days from first symptom)	233	8.5 ± 5.3	–
CRP (mg/l)	233	***123.0* ± *98.6***	0.2–10
Absolute WBC count (G/l)	233	8.9 ± 6.1	4.4–11.3
Absolute neutrophil count (G/l)	233	7.2 ± 7.7	2–8
Absolute lymphocyte count (G/l)	233	1.5 ± 4.3	0.8–4
Platelet count (G/l)	233	258.7 ± 108.3	150–400
PCT (ng/ml)	166	0.87 ± 7.40	0–0.5
LDH (U/l)	233	***744.7* ± *515.1***	230–460
D-dimer (ng/ml)	137	***2413.8* ± *4313.0***	0–500
ferritin (ng/ml)	124	***1207.4* ± *1927.4***	20–300
IL-6 (pg/ml)	67	***130.2* ± *138.4***	0–7
BUN (mmol/l)	233	6.6 ± 4.5	2.9–8.5
creatinine (μmol/l)	233	97.6 ± 89.9	64–104
Fever	233	146 (62.9)	–
Dyspnea	233	158 (68.1)	–
Coughs	233	162 (70.4)	–
Confusion/dizziness	233	10 (4.3)	–
PaO_2_ (mmHg)	199	58.4 ± 16.2	80–100*
SaO_2_ (%)	233	89.7 ± 7.8	95–99*
systolic BP (mmHg)	233	139.9 ± 23.5	90–140*
A-DROP	233	0.94 ± 0.79	0–1*
Immunosuppressive therapy (current)	233	17 (7.3)	–
Smoking (current)	68	13 (19.1)	–
**Medical history**	**Total *N***	***N* (%)**	
Hypertension (history)	233	151 (65.1)	–
CAD (history)	233	51 (22.0)	–
Stroke (history)	233	14 (6.0)	–
CKD (history)	233	13 (5.6)	–
Diabetes mellitus (history)	233	63 (27.2)	–
Obesity (history)	233	71 (30.6)	–
Malignancy (history)	233	10 (4.3)	–
COPD/asthma (history)	233	51 (22.0)	–
**Outcome measures**	**Total *N***	**Mean ± *SD* or *N* (%)**	
Time of hospitalization (days)	233	12.1 ± 6.8	–
ICU admission	233	49 (21.2)	–
Need for ventilation	233	46 (19.7)	–
Need for NIV	233	9 (3.9)	–
Need for IV	233	37 (15.9)	–
Deaths	233	40 (17.2)	–

*Age-dependent. Significantly elevated mean values are in bold italics. A-DROP, Age, Dehydration, Respiratory failure, Orientation disturbance (confusion) and low blood Pressure; BP, blood pressure; BUN, blood urea nitrogen; CAD, coronary artery disease; CTSS, CT chest Severity Score; CKD, chronic kidney disease; COPD, chronic obstructive pulmonary disease; CRP, C-reactive protein; ICU, intensive care unit; IL, interleukin; IV, invasive ventilation; LDH, lactate dehydrogenase; NIV, non-invasive ventilation; PaO_2_, partial oxygen pressure; PCT, procalcitonin; SaO_2_, oxygen saturation; WBC, white blood cell.

### Determinants of intensive care units admission, need for ventilation and death

In the binary analysis, admission to ICU was significantly more often associated with the duration of hospitalization (*p* < 0.001), hypertension (*p* = 0.002) or obesity (*p* = 0.014) in the medical history, as well as with confusion/dizziness at hospital admission (*p* = 0.034). Among the laboratory parameters, ICU admission was associated with higher absolute leukocyte (*p* = 0.045), higher neutrophil (*p* = 0.034) and lower lymphocyte counts (*p* = 0.007), CRP (*p* < 0.001), PCT (*p* < 0.001), LDH (*p* < 0.001), ferritin (*p* = 0.042), IL-6 (*p* = 0.026), BUN (*p* = 0.015), creatinine (*p* = 0.001), PaO_2_ (*p* = 0.001) and SaO_2_ (*p* = 0.001) (Table 2).

**TABLE 2:** Determinants of ICU admission, need for ventilation and survival.

Parameter	*p*-value		
	
	ICU vs. non-ICU	Vent vs. no vent	Death vs. survival
Age	0.121	0.078	***0.003***
Disease duration at admission	0.304	0.720	0.134
Duration of hospitalization	**<*0.001***	**<*0.001***	0.190
Male sex	0.050	***0.018***	0.097
Immunosuppressive therapy	0.261	0.588	0.589
Current smoker	0.154	0.221	0.326
Hypertension (history)	***0.002***	***0.010***	***0.011***
CAD (history)	0.929	0.691	***0.029***
Stroke (history)	0.599	0.649	0.206
CKD (history)	0.197	0.503	0.395
Diabetes mellitus (history)	0.182	0.239	***0.045***
Obesity (BMI)	***0.014***	***0.031***	***0.043***
Malignancy (history)	0.135	***0.021***	0.236
COPD/asthma (history)	0.929	0.931	0.354
Fever	0.292	0.560	0.309
Dyspnea	0.422	0.174	0.868
Coughs	0.885	0.120	0.846
Confusion/dizziness	***0.034***	***0.044***	***0.014***
Absolute WBC count	***0.045***	***0.012***	***0.007***
Absolute neutrophil count	***0.034***	***0.031***	**<*0.001***
Absolute lymphocyte count	***0.007***	***0.011***	***0.003***
Absolute platelet count	0.276	0.141	0.891
CRP	**<*0.001***	**<*0.001***	**<*0.001***
PCT	**<*0.001***	**<*0.001***	**<*0.001***
LDH	**<*0.001***	**<*0.001***	**<*0.001***
D-dimer	0.124	0.087	***0.009***
Ferritin	***0.042***	0.102	***0.041***
IL-6	***0.026***	***0.024***	***0.014***
BUN	***0.015***	***0.001***	**<*0.001***
Creatinine	***0.001***	***0.001***	**<*0.001***
PaO_2_	***0.001***	***0.001***	***0.004***
SaO_2_	***0.001***	**<*0.001***	***0.002***
Systolic BP	0.777	0.513	0.505
A-DROP	***0.002***	**<*0.001***	**<*0.001***

Mann-Whitney test was used. Significant differences are in bold italics. A-DROP, Age, Dehydration, Respiratory failure, Orientation disturbance (confusion) and low blood Pressure; BMI, body mass index; BP, blood pressure; BUN, blood urea nitrogen; CAD, coronary artery disease; CKD, chronic kidney disease; COPD, chronic obstructive pulmonary disease; CRP, C-reactive protein; ICU, intensive care unit; IL, interleukin; LDH, lactate dehydrogenase; PaO_2_, partial oxygen pressure; PCT, procalcitonin; SaO_2_, oxygen saturation; Vent, ventilation; WBC, white blood cell.

The need for ventilation was significantly associated with days of hospitalization (*p* < 0.001), male sex (*p* = 0.018), history of hypertension (*p* = 0.010), obesity (*p* = 0.031) or malignancy (*p* = 0.021), as well as with confusion/dizziness upon admission (*p* = 0.044). Among the laboratory parameters, the need for ventilation was associated with leukocytosis (*p* = 0.012), neutrophilia (*p* = 0.031) and lymphopenia (*p* = 0.011), as well as CRP (*p* < 0.001), PCT (*p* < 0.001), LDH (*p* < 0.001), IL-6 (*p* = 0.024), BUN (*p* = 0.001), creatinine (*p* = 0.001), PaO_2_ (*p* = 0.001), and SaO_2_ (*p* < 0.001) (Table 2).

Finally, death was associated with age (*p* = 0.003), hypertension (*p* = 0.011), CAD (*p* = 0.029), diabetes mellitus (*p* = 0.045) or obesity (*p* = 0.043) in the medical history, as well as with confusion/dizziness at hospital admission (*p* = 0.014). Poor survival was associated with higher absolute leukocyte (*p* = 0.007) and neutrophil (*p* < 0.001) but lower lymphocyte counts (*p* = 0.003), as well as CRP (*p* < 0.001), PCT (*p* < 0.001), LDH (*p* < 0.001), D-dimer (*p* = 0.009), ferritin (*p* = 0.041), IL-6 (*p* = 0.014), BUN (*p* < 0.001), creatinine (*p* < 0.001), PaO_2_ (*p* = 0.004) and SaO_2_ (*p* = 0.002) (Table 2).

We also assessed possible predictors of ICU admission and survival by ROC curve analysis. Again, higher age was significantly associated with mortality (*p* = 0.003), but not with the need for ICU admission (*p* = 0.121) (Table 3). Both lower arterial PaO_2_ and SaO_2_ were associated with increased need for ICU admission (*p* = 0.002 and *p* = 0.010, respectively) and death (*p* = 0.004 and *p* = 0.012, respectively) (Table 3).

**TABLE 3:** Determinants of ICU admission and death.

Parameter	ICU admission (Y/N)					Death (Y/N)				
		
	Cutoff	Sens.	1-Spec.	ROC area	*p*-value	Cutoff	Sens.	1-Spec.	ROC area	*p*-value
A-DROP	1.5	0.35	0.17	0.61 ± 0.05	***0.026***	1.5	0.47	0.16	0.71 ± 0.05	**<*0.001***
Age (year)	64.5	0.35	0.21	0.57 ± 0.05	0.121	61.5	0.58	0.32	0.65 ± 0.05	***0.003***
PaO_2_ (mmHg)	53.7	0.41	0.71	0.33 ± 0.05	***0.002***	55.3	0.32	0.63	0.34 ± 0.06	***0.004***
SaO_2_ (%)	90.2	0.41	0.68	0.36 ± 0.05	***0.010***	87.6	0.52	0.80	0.36 ± 0.06	***0.012***

ROC analysis was performed. Significant differences are in bold italics. A-DROP, Age, Dehydration, Respiratory failure, Orientation disturbance (confusion) and low blood Pressure; N, no; PaO_2_, partial arterial oxygen pressure; SaO_2_, oxygen saturation; Sens., sensitivity; Spec., specificity; Y, yes.

### A-DROP is a suitable method to assess general state and risk in COVID-19-associated pneumonia

**FIGURE 1:**
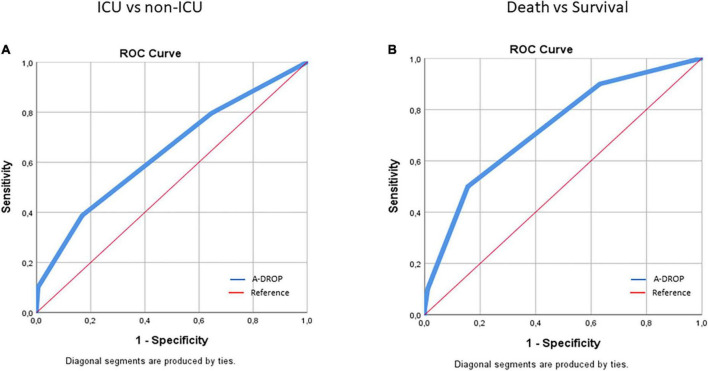
ROC curve analysis of the association of A-DROP values with the need for ICU admission **(A)** and death **(B)** in COVID-19 patients.

In the binary analysis, admission to ICU (*p* = 0.002), the need for ventilation (*p* < 0.001) and death (*p* < 0.001) were significantly associated with higher A-DROP (Table 2). In the ROC analysis, A-DROP > 1.5 significantly predicted admission to ICU (*p* = 0.026) and mortality (*p* < 0.001) (Table 3 and Figure 1). In the simple Spearman’s correlation analysis, A-DROP significantly and positively correlated with absolute WBC and neutrophil counts, CRP, PCT, LDH, D-dimer, ferritin, IL-6, and creatinine (Table 4).

**TABLE 4:** Correlations of A-DROP with clinical and laboratory parameters.

Parameter	A-DROP	
	
	*R*-value	*p*-value
Hospitalization days	0.097	0.159
Disease duration	0.129	0.057
Absolute WBC count	0.341	**<*0.001***
Absolute neutrophil count	0.339	**<*0.001***
Absolute lymphocyte count	–0.081	0.221
Absolute platelet count	0.043	0.514
CRP	0.270	**<*0.001***
PCT	0.599	**<*0.001***
LDH	0.299	**<*0.001***
D-dimer	0.354	**<*0.001***
Ferritin	0.421	**<*0.001***
IL-6	0.365	***0.002***
BUN	0.575	**<*0.001***
Creatinine	0.317	**<*0.001***
Systolic BP	0.065	0.331

Spearman’s correlation analysis was performed. Significant differences are in bold italics. A-DROP, Age, Dehydration, Respiratory failure, Orientation disturbance (confusion) and low blood Pressure; BP, blood pressure; BUN, blood urea nitrogen; CRP, C-reactive protein; IL, interleukin; LDH, lactate dehydrogenase; procalcitonin; WBC, white blood cell.

## Discussion

In this single-center study of 233 COVID-19 patients admitted to hospital, we assessed elements of medical history, as well as numerous clinical and laboratory parameters in association with the need for admission to ICU, need for ventilation and death. We also focused on the value of the A-DROP scoring system in the assessment of general health and prediction of outcome in hospitalized COVID-19 patients.

At the time of admission, among laboratory biomarkers, patients had elevated CRP, LDH, D-dimer, ferritin, and IL-6 levels. All these parameters, as well as higher absolute WBC and neutrophil and lower absolute lymphocyte counts, PCT, BUN, creatinine, PaO_2_, and SaO_2_ were associated with ICU admission, need for ventilation and death. Among clinical and other factor, age was associated with death only, male sex with the need for ventilation only and the duration of hospitalization with the need for ICU admission and ventilation. CRP, IL-6, ferritin, D-dimer, LDH and high neutrophil/lymphocyte, as well as BUN/creatinine ratios have been identified as markers of MIS/cytokine storm associated with SARS-CoV-2 infection (21–23). Both CRP and D-dimer levels were elevated in patients in need for transfer to ICU compared to non-ICU patients (22). D-dimer > 3,500 ng/ml was associated with poor survival (24). Obesity and confusion (dizziness) at the time admission, as well as the history of hypertension were associated with all three outcome measures. Obesity may be associated with increased mortality in COVID-19 (29). Dizziness has also been reported as an indicator of critical outcome in COVID-19 (30).

In addition to other known scoring systems, A-DROP has recently been validated for the assessment of health status in CAP (8, 25). In other studies, A-DROP has proven to be of great value in predicting CAP severity (8, 25). In the present cohort, the mean value of A-DROP at the time of admission was 0.94 on a 0–5 scale. A-DROP value of two or above were significantly associated with the need for ICU admission and ventilation, as well as with death. A-DROP also significantly correlated with absolute WBC and neutrophil counts, CRP, PCT, LDH, D-dimer, ferritin, IL-6, BUN, and creatinine. As discussed above, most of these parameters have been associated with severe COVID-19 including MIS and cytokine storm (21–23).

In other studies, various cardio-pulmonary, renal, hepatic, hematologic, and immunologic comorbidities have been associated with poor COVID-19 outcome (12–15). In addition, similarly to our findings, CRP, IL-6, ferritin, D-dimer, LDH, and troponin have been identified as severity and prognostic markers of COVID-19-associated MIS (21–23).

This study has certain strengths and limitations. The major strength of this study is that this is the first relatively large study assessing the prognostic value of A-DROP in a complex way, in association with numerous clinical and laboratory markers of outcome including ICU admission, ventilation and death in COVID-19. Possible limitations may include the single-center nature of the study. In addition, we have not included chest CT scans in this analysis, we have not validated our findings against other cohorts and we have not considered for population-specific biases.

## Conclusion

In conclusion, A-DROP may be a suitable scoring system for predicting the need for ICU admission and ventilation, as well as mortality in COVID-19. In our study, we identified several clinical and laboratory parameters that, when combined with the A-DROP scoring system, could further increase its sensitivity and specificity, providing clinicians with an appropriate risk assessment tool to identify high-risk patients in need of advanced health care. Further studies are planned to develop a scoring system with sufficient sensitivity and specificity.

## Data availability statement

The original contributions presented in the study are included in the article/supplementary material, further inquiries can be directed to the corresponding author/s.

## Ethics statement

The studies involving human participants were reviewed and approved by the Borsod Academic County Hospital (BORS 04/2021). The patients/participants provided their written informed consent to participate in this study.

## Author contributions

MS: study design, patient recruitment, data collection, manuscript drafting, and finalization. ZK, PT, CO, and EC: patient recruitment and data collection. KH: statistical analysis and data interpretation. ZS: supervisor, study design, manuscript drafting, and finalization. All authors contributed to the article and approved the submitted version.

## Conflict of interest

The authors declare that the research was conducted in the absence of any commercial or financial relationships that could be construed as a potential conflict of interest.

## Publisher’s note

All claims expressed in this article are solely those of the authors and do not necessarily represent those of their affiliated organizations, or those of the publisher, the editors and the reviewers. Any product that may be evaluated in this article, or claim that may be made by its manufacturer, is not guaranteed or endorsed by the publisher.
